# Melatonin and Expression of Tryptophan Decarboxylase Gene (*TDC*) in Herbaceous Peony (*Paeonia lactiflora* Pall.) Flowers

**DOI:** 10.3390/molecules23051164

**Published:** 2018-05-12

**Authors:** Daqiu Zhao, Rong Wang, Ding Liu, Yanqing Wu, Jing Sun, Jun Tao

**Affiliations:** 1 Jiangsu Key Laboratory of Crop Genetics and Physiology, College of Horticulture and Plant Protection, Yangzhou University, Yangzhou 225009, China; dqzhao@yzu.edu.cn (D.Z.); rongwang702@163.com (R.W.); liuding@yzu.edu.cn (D.L.); yqwu19880928@126.com (Y.W.); jingsun@yzu.edu.cn (J.S.); 2 Institute of Flowers and Trees Industry, Yangzhou University-Rugao City, Rugao 226500, China

**Keywords:** herbaceous peony, melatonin, flowers, gene expression, light exposure, tryptophan decarboxylase

## Abstract

Melatonin is a bioactive, edible ingredient that promotes human health and exists widely in plants, but little is known about its biosynthetic routes and underlying molecular mechanisms in the herbaceous peony. In this contribution, we found that herbaceous peony flowers are rich in melatonin that is found in the greatest quantities in the white series, followed by the ink series, the red series and then the pink series. On this basis, the melatonin content fluctuates during flower development and peaks during the bloom stage. Moreover, it is apparent that sun exposure and blue light induce melatonin production whereas green light restrains it during a 24-h light/dark cycle of melatonin content, as there were ‘dual peaks’ at 2 p.m. and 2 a.m. Additionally, the corresponding expression pattern of the herbaceous peony tryptophan decarboxylase gene (*TDC*) was positively related with melatonin production. These results suggest that color series, development stage and light play an important role in melatonin accumulation, and that *TDC* is a rate-limiting gene in melatonin biosynthesis.

## 1. Introduction

Melatonin (*N*-acetyl-5-methoxytryptamine) is an indole compound, which is conserved in the process of biological evolution and widely distributed in animals, plants and microorganisms [1]. In animals, melatonin can regulate the circadian rhythm [2] and act as a highly potent endogenous free radical scavenger that can effectively remove hydroxyl radicals, peroxy alkyl radicals, hydrogen peroxide, superoxide anion radicals and singlet oxygen [3,4]. Moreover, melatonin has been proved to have a number of physiological functions in humans including enhancing immunity, anti-tumor effects, anti-aging effects, and in particular, it has effects on Alzheimer’s disease [5]. Since its first discovery in plants in 1995 [6], melatonin has been found in almost all plants, and numerous studies have confirmed that melatonin functions as an important reactive oxygen species (ROS) scavenger in plants as well as in animals [7,8,9]. 

As melatonin is natural and has potent antioxidant activity, it is now being evaluated as a bioactive, edible ingredient to promote human health. Garrido et al. [10] found that the melatonin in food could be absorbed by the digestive tract to then play a physiological role in the body. When people eat fruits and vegetables rich in melatonin, the levels of melatonin secretion in the body increase, thus improving the antioxidant capacity of the body and effectively improving immune regulation function [9]. Melatonin exhibits a wide variety of levels in plants, but in most fruits and vegetables, including *Actinidia chinensis, Cucumis sativus, Musa acuminata*, *Malus domestica* and *Fragaria* × *ananassa*, fruit tissue melatonin concentrations are less than 50 pg/g fresh weight (FW), which is a very low level [11], whereas medicinal plants contain a high level of melatonin, and traditional medicinal herbs with high melatonin content have the effect of delaying senescence and performing antioxidation functions [12]. 

The synthetic pathway of melatonin in animals has been thoroughly studied but this pathway needs to be further documented in plants. The key enzymes involving melatonin biosynthesis have been detected in plants, including l-tryptophan decarboxylase (TDC) [13,14], tryptophan hydroxylase (T5H) [15,16], serotonin-*N*-acetyltransferase (SNAcT) [17], *N*-acetylserotonin methyltransferase (ASMT) [18,19] and hydroxyindole-*O*-methyltransferase (HIOMT) [20]. TDC belongs to the group of aromatic-l-amino acid decarboxylases, and it is also known as the first rate-limiting enzyme in the melatonin synthesis pathway [13,14]. The *TDC* gene has been identified in several plant species, but its importance in plant melatonin synthesis remains to be determined [21,22].

The herbaceous peony (*Paeonia lactiflora* Pall.) belongs to the Paeoniaceae family and has been cultivated for more than 4000 years in China. As the king of herbaceous flowers, *P. lactiflora* is widely used in urban landscaping, courtyards, special gardens, etc. Aside from its ornamental value, the whole plant is precious and has high medicinal value as its flowers and roots can be used as medicine [23]. In recent years, the edible value of *P. lactiflora* has also received more attention. *P. lactiflora* flowers can be processed into teas, nectar and porridge, and the root can be made into soup. Shang et al. [24] found that *P. lactiflora* flowers contain abundant protein, sugar, phenols, ascorbic acid, mineral elements and important fatty acids, including palmitic acid and linoleic acid. Jin et al. [25] also confirmed that *P. lactiflora* flowers contain carbohydrates, glycosides, organic acids, flavonoids, phenols, and terpenoids, while the extract of *P. lactiflora* flowers can effectively clear active oxygen free radicals. Meanwhile, we detected melatonin in *P. lactiflora* flowers, leaves, and roots, and the highest level was found in flowers, with 62.61 ± 3.23 ng/g FW, which was as high as 1.96 and 5.03 times of those measured in the roots and leaves, respectively [26]. Accordingly, *P. lactiflora* flowers are rich in nutrients and antioxidant components, which has important significance in food and healthcare research and development. Currently, research into plant melatonin is in an exponential growth phase, and a large volume of research has been focused on the involvement of melatonin in aspects of plant development regulation and the function of melatonin as an alleviating-stressor agent [27,28,29]. However, little is known about melatonin’s metabolic pathways or the rate-limiting enzyme for its synthesis in high plants. To explore the biosynthesis of melatonin, the melatonin content of four color series *P. lactiflora* cultivars was measured. On this basis, we detected the melatonin levels produced during flower development under different light and temperature conditions; moreover, the corresponding expression patterns of the *TDC* gene in the melatonin synthesis pathway were monitored to characterize the specific function of *TDC* in *P. lactiflora* melatonin biosynthesis. These results provide novel information on melatonin synthesis and could contribute to an improvement in the production of melatonin in *P. lactiflora*.

## 2. Results

The melatonin content of different cultivars varies greatly which is largely related to the genotype of cultivars. To identify the rhythm associations of melatonin production with color in *P. lactiflora*, sixteen cultivars involved in four color series were selected for this study. First, the *H°* value was used to describe the color of the flowers: 0° means red and purple, 90° means yellow, 180° means blue-green, and 270° is blue. The *H°* of the white series was between 106.7–117.6°, the pink series was between 19.5–56.5°, the red series was between 348.1–357.9° and the ink series was between 0.8–6.5°. In Table 1, the overall trend of melatonin content in different color series flowers was shown in the order of white series > ink series > red series > pink series. The average melatonin content in the ink series and white series was more than 5 ng/g FW, and the white series had the highest melatonin content, reaching 5.77 ng/g FW, while the pink series had the lowest content with only 4.23 ng/g FW. Moreover, the melatonin content of ‘Xue Feng’ in the white series was the highest, reaching 5.97 ± 0.19 ng/g FW, while the content of ‘Fenchi Dizhi’ in the pink series was the lowest, at only 66.09% of ‘Xue Feng’.

**Figure 1:**
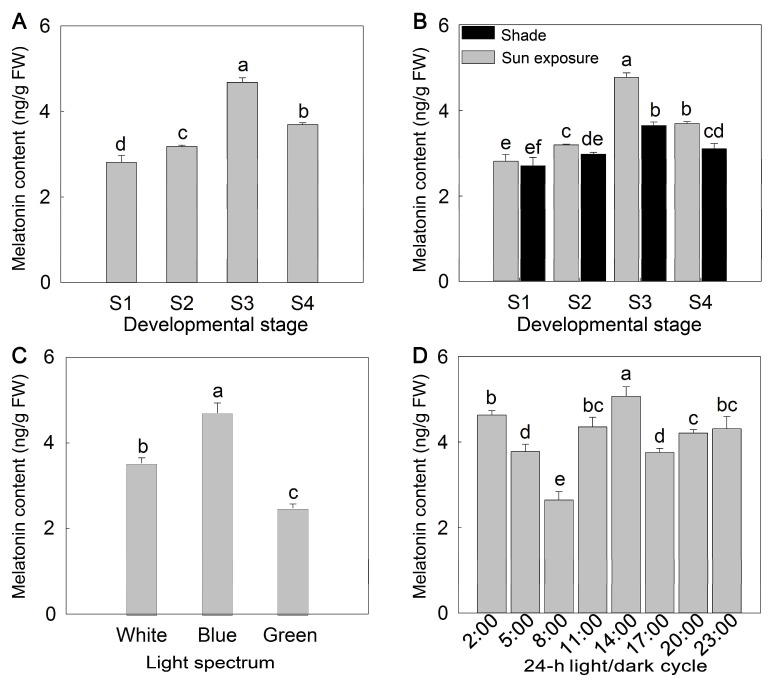
Changes in melatonin content in *P. lactiflora* ‘Zi Fengyu’ flowers. (**A**) Effect of the flower developmental stage on the melatonin content in *P. lactiflora* flowers. (**B**) Effect of shade on the melatonin content in *P. lactiflora* flowers. (**C**) Effect of different light spectrums on the melatonin content in *P. lactiflora* flowers. (**D**) Effect of the 24-h light/dark cycle on the melatonin content in *P. lactiflora* flowers. S1, flower-bud stage; S2, initiating bloom; S3, bloom stage; S4, wither stage. The values represented the means ± SDs, and different letters indicate significant differences according to Duncan’s multiple range test (*p <* 0.05).

**Figure 2:**
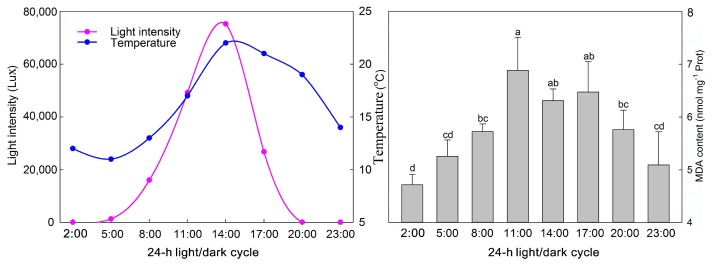
Changes in light intensity, temperature and malonyldialdehyde (MDA) content during the 24-h light/dark cycle. The values represented the means ± SDs, and different letters indicate significant differences according to Duncan’s multiple range test (*p* < 0.05).

Melatonin content is closely related to the flower developmental stage and light exposure. To identify the rhythmic associations of melatonin production with developmental stage and light in *P. lactiflora*, flowers under different treatments were used for melatonin content measurement. As shown in Figure 1A, during the development of flowers, the melatonin content first increased, then decreased, and then peaked in S3 where the value was 4.68 ± 0.10 ng/g FW. The value in S1 was the lowest with only 2.81 ± 0.16 ng/g FW; therefore, the value in S1 was only 60% of S3. The melatonin content in S4 was a little higher than the value in S2 (Figure 1A). Under sun exposure and shade conditions, the melatonin content of flowers at different developmental stages also increased first and then decreased, the content in S1 was the lowest and that in S3 was the highest, so the trend was basically similar. The melatonin content was always higher during sun exposure than under shade conditions. The difference between these conditions was the smallest in S1, which was 0.10 ng/g FW and the biggest difference between them was in S3 with a difference of 1.12 ng/g FW (Figure 1B). For the light spectrum, it was apparent that blue light induces melatonin production whereas green light restrains it, and a significant difference was reached between the different light spectrum treatments. The highest melatonin content occurred under blue light, with 4.70 ± 0.24 ng/g FW, which was about 33% and 90% higher than the content measured under white light and green light, respectively (Figure 1C). In a 24-h light/dark cycle, the highest light intensity and temperature occurred at 14:00, and two malonyldialdehyde (MDA) peaks occurred at 11 a.m. and 5 p.m. (Figure 2). Regarding changes in melatonin content, there were also ‘dual peaks’ at 2 p.m. and 2 a.m., and the lowest content was measured at 8 a.m., which was only 52.12% of that measured at 2 p.m. (Figure 1D).

**Figure 3:**
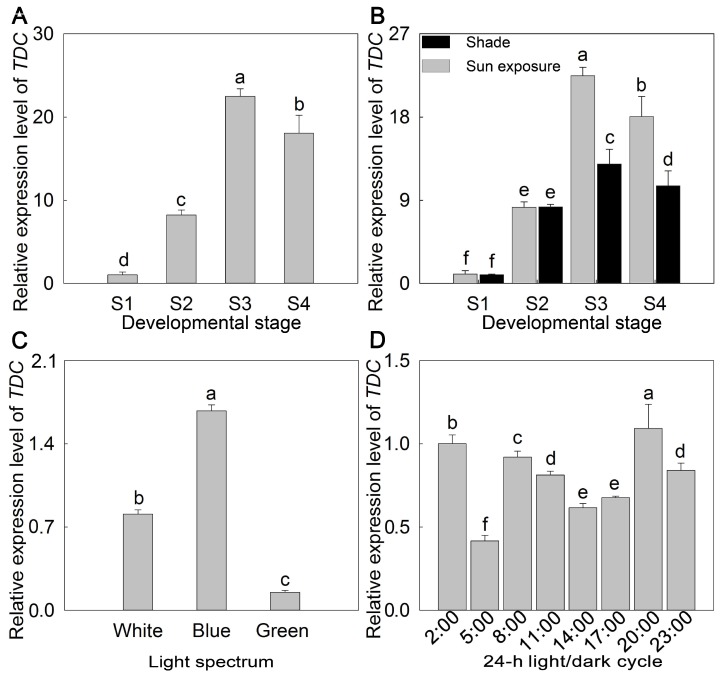
Changes in the relative expression level of *TDC* in *P. lactiflora* ‘Zi Fengyu’ flowers. (**A**) Effect of the flower developmental stage on the relative expression level of *TDC* in *P. lactiflora* flowers. (**B**) Effect of shade on the relative expression level of *TDC* in *P. lactiflora* flowers. (**C**) Effect of different light spectrums on the relative expression level of *TDC* in *P. lactiflora* flowers. (**D**) Effect of the 24-h light/dark cycle on the relative expression level of *TDC* in *P. lactiflora* flowers. S1, flower-bud stage; S2, initiating bloom; S3, bloom stage; S4, wither stage. The values represented the means ± SDs, and different letters indicate significant differences according to Duncan’s multiple range test (*p <* 0.05).

The melatonin biosynthetic pathway involves several genes [30], but only the *TDC* gene (NCBI Acc. No KY765554) was retrieved for *P. lactiflora*. In this study, the expression trend of the *TDC* gene was basically the same as that of melatonin content. During the development of flowers, the expression level of *TDC* presented a decreasing trend. The relative expression level of *TDC* in S3 was significantly higher than those in S1 and S2, and *TDC* was abundantly expressed in S3, at up to 21 times that of S1. Meanwhile, the *TDC* value was higher in S4 than in S2 (Figure 3A). Likewise, *TDC* expression and melatonin production were similarly affected by shade treatment. *TDC* expression was first increased and then decreased, similar to the effect of shade treatment on melatonin production. Meanwhile, expression under the sun exposure condition was higher than that under shade condition at the same stage (Figure 3B). Similarly, for the light spectrum, *TDC* expression was induced in blue light while restrained in green light. Its expression level under blue light was 8.5 times that under green light (Figure 3C). Additionally, *TDC* was expressed most heavily between 8 and 11 a.m. and 8 p.m. and 2 a.m., which preceded the melatonin changes in the 24-h light/dark cycle. However, the expression of *TDC* at 5 a.m. was the lowest (Figure 3D).

## 3. Discussion

Melatonin is an amphiphilic bioactive molecule that is found in a variety of plant species, in which the melatonin level can vary greatly depending on plant cultivars. González-Gómez et al. [31] determined the melatonin content of eight *Prunus avium* cultivars and found that it was significantly distinct in different varieties, where the content of ‘Burlat’ was the highest at 0.22 ng/g FW, while that of ‘Ambrunés’ was lower than the detection range. Stürtz et al. [32] found that the *Lycopersicon esculentum* cultivar ‘Marbone’ (114.5 ng/g FW) had 28 times the melatonin level of ‘Catalina’ (4.1 ng/g FW), and the *F. × ananassa* cultivar ‘Festival’ (11.26 ng/g FW) had eight times the amount of melatonin of ‘Camarosa’ (1.4 ng/g FW). These differences were mainly due to the specific plant genotype. In the present study, the diversity in melatonin content of flowers was also found in sixteen *P. lactiflora* cultivars, which had a certain correlation with color series and showed an overall trend: white > ink > red > pink series. The average melatonin content in the flowers of four white *P. lactiflora* cultivars reached 5.78 ng/g FW, while that in the pink cultivars was 4.23 ng/g FW. The flower color diversity of *P. lactiflora* reflects flavonoid accumulation [33] as flavonoids are a kind of important secondary metabolite in plants that have strong antioxidant activity [34]. The most prominent function of melatonin in plants is also antioxidation and the scavenging of free radicals, and melatonin treatment can increase flavonoid content [35]. However, the association between flavonoids and melatonin requires more research on this basis as the white series *P. lactiflora* flowers which have a high melatonin content could be developed for edible and medicinal uses.

The flower developmental stage affects the melatonin content in plants. *Datura metel* melatonin levels were found to be the highest in the youngest flower buds, and concentrations decreased as the flower buds matured [36]. However, during the ripening of *P. avium*, melatonin was found at the highest levels in the ripest fruit, whereas no quantifiable amounts or very low amounts were found in the other fruit ripening stages [31]. In this study, we reported that the melatonin content in *P. lactiflora* flowers increased from S1 (2.81 ng/g FW) to S3 (4.68 ng/g FW) with the ripening of flowers, and decreased in the wither stage (S4), which was consistent with observations in *P. avium* fruit [31] and was in contrast to *D. metel* flowers [36]. These results also suggest that the accumulation trend of melatonin is not consistent with the development stages of different plants. Consequently, further detailed study on melatonin biosynthesis in the flower developmental stages in plants is needed. Moreover, the melatonin content in plants is influenced by the growth environment [37], including the light intensity, light spectrum, and photoperiod, which play important roles in its accumulation [38,39]. Under shade conditions, the melatonin content in most of the *Capsicum annuum* cultivars decreased by 64% [40]. A high melatonin content was detected in *Oryza sativa* leaves under constant light, whereas the melatonin concentration was very low in constant darkness. The melatonin intermediates, tryptamine, serotonin and *N*-acetylserotonin, all decreased significantly in the darkness compared to the sunlight conditions [38]. Our results were in agreement with previous reports, in which the melatonin content of plants grown under shade was always lower the content in plants under sun exposure during flower development. These findings suggest that melatonin biosynthesis is dependent on light signals in *P. lactiflora* flowers. Furthermore, Afreen et al. [39] found that the melatonin concentrations in *Glycyrrhiza uralensis* varied depending on the light spectra in the following order: red >> blue ≥ white light. Zhang et al. [41] found that a light regime with a proportion of blue light could increase the melatonin content in milk. In our study, blue light also increased the concentration of melatonin in *P. lactiflora*, whereas green light decreased its concentration, suggesting that in *P. lactiflora*, melatonin biosynthesis was responsive to different light spectra. In addition, melatonin production responded to the circadian changes of the photoperiod and often produced the ‘dual peaks’ in plants [42]. In the current study, we also observed two peaks in *P. lactiflora* melatonin production in the 24-h light/dark cycle. The first peak of melatonin production at 2 a.m. was very similar to that observed in *Vitis vinifera* fruit, which was induced by darkness [43]. The second melatonin peak was at 14 p.m., when the ambient temperature and light intensity were the highest which could induce a large amount of ROS. In plants, both exogenously-applied and endogenously-produced melatonin could scavenge the overproduction of ROS [42,44,45]. Therefore, the second melatonin peak could be aimed at alleviating damage from high temperatures and intensive sunlight in *P. lactiflora* flowers. The detected MDA content confirmed the protective effects of melatonin against high temperatures and intensive sunlight in *P. lactiflora* flowers. Its peak value was observed at 11 a.m., well in advance of the melatonin peak (2 p.m.). This result may be because that the high temperature and light stresses (as indicated MDA levels) triggered melatonin synthesis and led to the second peak of melatonin at 2 p.m., consistent with that reported by Zhao et al. [45]. However, further research is needed to prove this.

Recently, there has been a growing interest in metabolic engineering aimed at increasing melatonin levels in food-producing plants [46,47]. As the first rate-limiting gene for melatonin biosynthesis in plants [45], melatonin and melatonin intermediate levels were greatly enhanced in *O. sativa* seeds through the overexpression of the *TDC3* (GenBank Accession Number: NM001067504) gene and a homozygous *TDC3* line (*TDC3-1*) had melatonin concentrations 31-fold higher than those of wild-type seeds [48]. To further understand the role of *TDC* in *P. lactiflora* melatonin biosynthesis, its expression pattern was analyzed. Zhao et al. [45] found that *TDC* expression was positively related to melatonin production in *P. avium*, and we also observed this positive correlation in our study. The relative expression level of *TDC* first increased and then decreased during *P. lactiflora* flower development, with the peak of its expression in S3, which was consistent with the melatonin content peak at the same developmental stage. Under the shade growth condition, a very low *TDC* level was observed, while a higher level was detected in rice leaves under constant light [38]. In *P. lactiflora* flowers, the relative expression level of *TDC* was also lower in shade-treated plants than in those grown under sun exposure, in agreement with the trends shown for melatonin. Furthermore, it was found that blue light induced *P. lactiflora* melatonin production and *TDC* expression, whereas green light inhibited the production/expression of both factors. In *Camptotheca acuminata*, blue light also significantly induced *TDC* expression and its enzymatic activity [49]. In a 24-h light/dark cycle, *P. lactiflora TDC* was expressed most heavily between 8 a.m. and 11 a.m. and 8 p.m. and 2 a.m., while the melatonin peaks occurred between 11 a.m. and 2 p.m. and between 11 p.m. and 2 a.m., lagging behind *TDC* expression. This might be because *TDC* was the first biosynthetic gene for melatonin biosynthesis. The positive relationship between *TDC* expression and melatonin production suggests that *TDC* is a key gene in melatonin biosynthesis in *P. lactiflora*. Therefore, the upregulation of *TDC* should lead to melatonin production in *P. lactiflora*.

In the present study, we researched the associations of melatonin production with color in *P. lactiflora*, detected the melatonin levels produced during flower development in different light and temperature conditions, and explored the roles of the *TDC* gene in melatonin production in *P. lactiflora* plants. We found that *P. lactiflora* flowers in white series were rich in melatonin. On this basis, white series *P. lactiflora* flowers could be developed for edible and medicinal uses. In addition, sun exposure and blue light induced melatonin production. At the same time, we found that the *TDC* gene was upregulated in sun exposure and blue light. Future studies and genetic analyses, together with the analysis of melatonin levels in *P. lactiflora* during a natural day/night cycle will shed light on the regulation of melatonin biosynthesis and thus, its possible functions.

## 4. Materials and Methods

### 4.1. Plant Materials and Treatments

*P. lactiflora* flowers were collected from the germplasm repository of the Horticulture and Plant Protection College, Yangzhou University, Jiangsu Province, China (32°30′ N, 119°25′ E). Four different color series of *P. lactiflora* blooming flowers were used to determinate the melatonin content, including white series (‘Xueshan Hongxing’, ‘Xueshan Hongmei’, ‘Yangfei Chuyu’ and ‘Xue Feng’), pink series (‘Zhongsheng Fen’, ‘Zhu Shapan’, ‘Fenchi Dizhi’, ‘Fenzhu Pan’ and ‘Dadi Lushuang’), red series (‘Dadi Lushuang’, ‘Da Fugui’, ‘Zi Fengyu’ and ‘Hong Feng’) and ink series (‘Heihai Botao’, ‘Moyun Hanjin’, ‘Molou Jinhui’ and ‘Yanzi Xiangyang’). Moreover, to identify the rhythm associations of melatonin content and *TDC* expression, *P. lactiflora* ‘Zi Fengyu’ flowers were collected at different developmental stages, as well as from plants grown under different growing conditions including shade, different light spectra and during a 24-h light/dark cycle. Flowers of different developmental stages were picked between April and May 2016. Four developmental stages were used: flower-bud stage (Stage 1, S1), initiating bloom stage (Stage 2, S2), bloom stage (Stage 3, S3) and wither stage (Stage 4, S4). For experiments conducted in March 2016 in shaded conditions, when *P. lactiflora* buds emerged, plants were covered with a black shade net that had 60% transmittance. Then, flowers of different developmental stages were harvested. For the light experiment, when *P. lactiflora* was at the flower-bud stage, the uniform flowers were cut and immediately stood upright into buckets partially filled with deionized water. After being transported to our laboratory, the stem-ends were cut crosswise under the deionized water into approximately 30 cm in length with two compound leaves. Then, these flowers were all inserted into a 2% sucrose solution and transferred together to a room at 25 °C and 60% relative humidity under white, blue or green light. After 48 h, flowers were picked without stems. For the 24-h light/dark cycle experiment, flowers in a field were picked at eight points during a 24-h light/dark cycle (2 a.m., 5 a.m., 8 a.m., 11 a.m., 2 p.m., 5 p.m., 8 p.m., and 11 p.m. on 28 April, 2016) when *P. lactiflora* was at the bloom stage. All samples were immediately frozen in liquid nitrogen and stored at −80 °C until further melatonin content measurement and *TDC* expression analysis.

### 4.2. Color Indices Measurement

The color of fresh petals was firstly compared with the RHSCC [50]. Then, the color indices were measured with a hand-held RM200QC spectrocolorimeter (X-Rite, Grand Rapids, MI, USA.) using two color parameters including a* and b* values. The hue angles (*H°* = arctangent (b*/a*)) were calculated.

### 4.3. Melatonin and MDA Content Measurement

Before the melatonin content was measured, the samples were pre-treated. First, 0.1 g of sample was ground into a fine powder with liquid nitrogen and extracted with 1.0 mM phosphate buffer (pH 7.2 and containing 5% methanol) in a 1.5 mL centrifuge tube. Second, the mixed sample was moved on ice and ultrasonicated at 100 W for 30 s using an ultrasonic instrument (VCX-130, Sonics, Newtown, CT, USA), and then the extract was centrifuged at 4 °C for 10 min at 10,000× *g* and the resulting supernatant was collected. Subsequently, the melatonin was detected according to the guidelines of an ELISA kit (Shanghai Qiaodu Biotechnology Co., Ltd., Shanghai, China), and its content was determined by the SpectraMax M5 plate reader (Molecular Devices Corporation, Sunnyvale, CA, USA). The MDA content of petals was determined by the thiobarbituric acid (TBA) method using reagent kits from the Nanjing Jiancheng Bioengineering Institute, China.

### 4.4. Gene Expression Analysis

Gene transcript levels were analyzed using real-time quantitative polymerase chain reaction (qRT-PCR) with a BIO-RAD CFX ConnectTM Optics Module (Bio-Rad, Des Plaines, IL, USA). Total RNA was extracted according to a modified CTAB extraction protocol [51], and its integrity was checked by a spectrophotometer (Eppendorf, Hamburg, Germany). The cDNA was synthesized from 1 µg RNA using a PrimeScript^®^ RT reagent kit with a gDNA Eraser (TaKaRa, Tokyo, Japan). In this study, *P. lactiflora Actin* (NCBI Acc. No. JN105299) (forward primer: 5′-ACTGCTGAACGGGAAATT-3′, reverse primer: 5′-ATGGCTGGAACAGGACTT-3′ was used as internal control [33], and the *Actin* primer set amplified the expected fragment of *Actin* gene consisting of 187 bp. Gene-specific primers of *P. lactiflora TDC* (NCBI Acc. No. KY765554) were as follows: forward primer: 5′-GTTGGGTGACACGGAAAC-3′, reverse primer: 5′-GACCGCAAATCTCAGCAT-3′). The *TDC* primer set amplified the expected fragment of *TDC* gene consisting of 115 bp. qRT-PCR was performed using the SYBR^®^ Premix Ex Taq^TM^ (Perfect Real Time) (TaKaRa, Japan) and contained 12.5 µL 2 × SYBR Premix Ex Taq^TM^ buffer, 2 µL cDNA solution, 2 µL mix solution of target gene primers (10 µM) and 8.5 µL ddH_2_O in a final volume of 25 µL. The amplification was carried out under the following conditions: 95 °C for 30 s, 40 cycles at 95 °C for 5 s, 52 °C for 30 s, and 72 °C for 30 s. The relative expression levels of target genes were calculated by the 2^−^^△△Ct^ comparative threshold cycle (Ct) method [52]. The Ct values of the triplicate reactions were gathered using the Bio-Rad CFX Manager V1.6.541.1028 software (Bio-Rad, Des Plaines, IL, USA).

### 4.5. Statistical Analysis

All experiments described here were repeated three times in a completely randomized design. Primers were designed using the Primer 5.0 program (Premier Biosoft, Palo Alto, CA, USA). All data presented are the means of three replicates with standard deviations. The results were analyzed for variance using the SAS/STAT statistical analysis package (version 6.12, SAS Institute, Cary, NC, USA).

## Figures and Tables

**Table 1:** Melatonin content of different color series *P. lactiflora* cultivars.

Color	Cultivars	Flower	RHSCC	*H°*	Melatonin (ng/g FW)
Pink Series	‘Zhongsheng Fen’	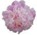	69 A	20.4	4.31 ± 0.36 ^f^
	‘Zhusha Pan’	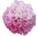	69 B	56.5	4.35 ± 0.13 ^f^
	‘Fenchi Dizhi’	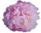	69 B	19.5	3.94 ± 0.11 ^g^
	‘Fen Zhupan’	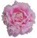	69 B	38.5	4.34 ± 0.07 ^f^
Red Series	‘Dadi Lushuang’	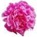	72 A	348.7	4.81 ± 0.22 ^d,e^
	‘Da Fugui’	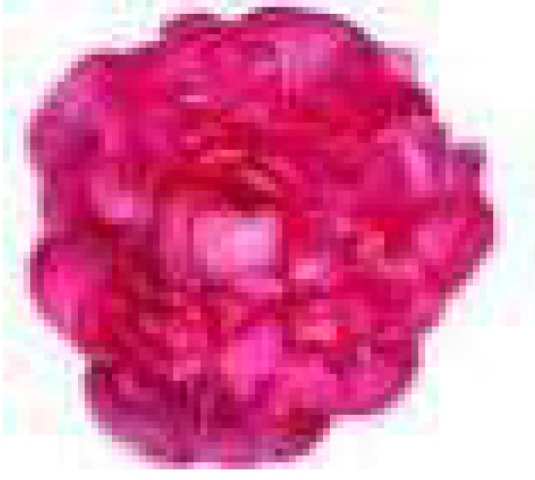	72 A	348.1	4.74 ± 0.16 ^e^
	‘Zi Fengyu’	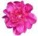	71 A	357.9	4.82 ± 0.10 ^d,e^
	‘Hong Feng’	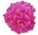	71 C	349.7	4.73 ± 0.08 ^e^
Black ink Series	‘Heihai Botao’	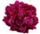	59 A	1.7	5.25 ± 0.13 ^c^
	‘Moyun Hanjin’	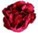	N77 A	3.7	4.97 ± 0.05d ^e^
	‘Molou Jinhui’	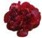	59 A	0.8	5.26 ± 0.13 ^c^
	‘Yanzi Xiangyang’	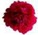	59 B	6.5	5.05 ± 0.08 ^c,d^
White Series	‘Xueshan Hongxing’	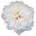	155 B	117.6	5.59 ± 0.29 ^b^
	‘Xueshan Hongmei’	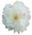	155 B	120.2	5.75 ± 0.14 ^a,b^
	‘Yangfei Chuyu’	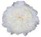	NN155 B	110.5	5.80 ± 0.21 ^b^
	‘Xue Feng’	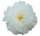	NN155 B	106.7	5.97 ± 0.19 ^a^

The values represented the means ± SDs, and different letters indicate significant differences according to Duncan’s multiple range test (*p <* 0.05). RHSCC: Royal Horticultural Society color chart; *H°:* chromaticity angle.
